# NAFLD and HBV interplay - related mechanisms underlying liver disease progression

**DOI:** 10.3389/fimmu.2022.965548

**Published:** 2022-12-05

**Authors:** Evanthia Tourkochristou, Stelios F. Assimakopoulos, Konstantinos Thomopoulos, Markos Marangos, Christos Triantos

**Affiliations:** ^1^ Division of Gastroenterology, Department of Internal Medicine, Medical School, University of Patras, Patras, Greece; ^2^ Division of Infectious Diseases, Department of Internal Medicine, Medical School, University of Patras, Patras, Greece

**Keywords:** NAFLD, HBV, infection, hepatic steatosis, HCC, liver disease

## Abstract

Non-alcoholic fatty liver disease (NAFLD) and Hepatitis B virus infection (HBV) constitute common chronic liver diseases with worldwide distribution. NAFLD burden is expected to grow in the coming decade, especially in western countries, considering the increased incidence of diabetes and obesity. Despite the organized HBV vaccinations and use of anti-viral therapies globally, HBV infection remains endemic and challenging public health issue. As both NAFLD and HBV have been associated with the development of progressive fibrosis, cirrhosis and hepatocellular carcinoma (HCC), the co-occurrence of both diseases has gained great research and clinical interest. The causative relationship between NAFLD and HBV infection has not been elucidated so far. Dysregulated fatty acid metabolism and lipotoxicity in NAFLD disease seems to initiate activation of signaling pathways that enhance pro-inflammatory responses and disrupt hepatocyte cell homeostasis, promoting progression of NAFLD disease to NASH, fibrosis and HCC and can affect HBV replication and immune encountering of HBV virus, which may further have impact on liver disease progression. Chronic HBV infection is suggested to have an influence on metabolic changes, which could lead to NAFLD development and the HBV-induced inflammatory responses and molecular pathways may constitute an aggravating factor in hepatic steatosis development. The observed altered immune homeostasis in both HBV infection and NAFLD could be associated with progression to HCC development. Elucidation of the possible mechanisms beyond HBV chronic infection and NAFLD diseases, which could lead to advanced liver disease or increase the risk for severe complications, in the case of HBV-NAFLD co-existence is of high clinical significance in the context of designing effective therapeutic targets.

## Introduction

Non-alcoholic fatty liver disease (NAFLD) constitutes the most common chronic liver disease, affecting approximately 25% of adults globally (1). The increased incidence of diabetes and obesity in western countries, seems to contribute to the growth of NAFLD burden in the coming decade (2) considering that NAFLD has been associated with metabolic syndrome and insulin resistance (3). A spectrum of liver disease stages and complications have been attributed to NAFLD, including simple steatosis, non-alcoholic steatohepatitis (NASH), progressive fibrosis, and hepatocellular carcinoma (HCC) (4). Hepatitis B virus infection (HBV) plays also a major causative role in the development of liver pathologies, such as cirrhosis and hepatocellular carcinoma and leads to increased liver-related mortality and morbidity (5, 6). Although the worldwide uptake of HBV vaccinations may have restrained HBV transmission, HBV remains endemic and challenging public health issue, especially in low- and middle-income countries. According to World Health Organization 296 million people worldwide were estimated to live with chronic HBV infection and 820000 HBV-related deaths, mainly from cirrhosis and HCC, were reported in 2019, with 1.5 million new infections being reported each year (7). The co-occurrence of NAFLD and HBV infection has gained great research and clinical interest, regarding the chronic liver injury progression to severe complications under the effect of both diseases. Biopsy-proven NAFLD has been estimated in Chronic hepatitis B (CHB) patients to range from 14% to 30% (8, 9). Investigation of the relationship between CHB and NAFLD disease is still ongoing. Hepatic steatosis may have a favorable effect on CHB progression, by accelerating HBsAg serum clearance (10). In contrast, the co-occurrence of chronic HBV infection and NAFLD has been associated with increased risk for advanced liver disease and HCC (11). NAFLD has been remarked as causative agent of elevated ALT enzyme with a rate of 25%, in CHB patients (12). HBV has been shown to increase the risk for hepatic steatosis *in vivo* and specifically HBx protein has been proved to upregulate the liver fatty-acid binding proteins, promoting hepatic lipid accumulation (13). However, clinical studies have reported that only metabolic factors are independently associated with NAFLD (14). The management of patients with CHB and NAFLD post a new challenge in clinical practice, considering that little is known about the possible interaction of two liver pathologies and the pathologic outcomes of their interaction. Thus, in this review we aim to describe the possible mechanisms beyond HBV chronic infection and NAFLD diseases, which could lead to advanced liver disease or increase the risk for severe complications, in the case of HBV-NAFLD co-existence.

## Possible effects of NAFLD on liver disease progression during HBV infection

The causative relationship between NAFLD and HBV infection has not been elucidated so far. Metabolic components and immune alterations which are related to NAFLD progression have been suggested to directly inhibit HBV replication or induce indirectly anti-viral immune responses. A significant increase in Th17 cell related gene expression, including cytokine IL-21, has been remarked in NASH patients (15), which may contribute to HBV clearance. IL21 levels have been found elevated and positively associated with HBV DNA and HBeAg in immune clearance phase of chronic HBV infection, compared to immune tolerance phase (16). Increased serum levels of IL-21 in HBV-related liver failure may contribute to activation of T and B cells, which will produce inflammatory cytokines and eliminate virus proliferation and subsequent liver injury. Thus, persistence of HBV infection could be probably attributed to low levels of IL-21 (17, 18).

Elevated expression of Toll-like receptors (TLRs) has been remarked in NASH stage (19, 20), which is accompanied by increased infiltration and activation of adaptive immune cells, such as CD8+ T cells and NKT cells (21). TLRs play a major role in activation and modulation of immune responses and their activity has been highlighted in the pathogenesis and progression of chronic liver diseases, including HBV and HCV infection, alcoholic liver disease, hepatic fibrosis, NAFLD/NASH, cirrhosis and hepatocellular carcinoma (22–24). TLRs are highly distributed in liver cells [hepatocytes, kupffer cells (KCs), hepatic stellate cells (HSCs), sinusoidal endothelial cells, hepatic dendritic cells (DCs)] and many other liver cell populations can respond to TLRs. TLR signaling contributes to chronic liver disease progression, by mediating inflammatory processes and liver pathologies (e.g. hepatocellular injury and regeneration, fibrosis and cirrhosis) (25). Stimulation of TLRs in HSCs, upon activation of pro-inflammatory IKK/NF-κβ signaling, c-Jun N-terminal kinase (JNK) activity and secretion of pro-inflammatory cytokines and chemokines, leads to hepatic stellate cell activation and differentiation, promoting fibrosis (26–29). TLR5 has been shown to have an impact on the progression of fibrosis, by activating NF-κB and MAPK signaling pathways (30). Activation of the NF-κB and JNK pathways have been associated with production of cytokines related to TLR-induced liver damage and HCC progression (24). Although the activation of adaptive immune cells in NASH (21) may enhance further the anti-viral immune responses in HBV-infection and prevent the HBV-related severe liver disease progression, the increased expression and activity of TLRs in NASH stage of NAFLD may aggravate liver disease progression to fibrosis and HCC.

TLRs are activated in recruited hepatic DCs in liver sinusoids during inflammation and induce production of pro-inflammatory cytokines (TNF-α, IL-6, IL-12) (31, 32). Saturated fatty acids, have been shown to induce TLR4 activation and activate immune responses through myeloid differentiation factor 88 (MyD88)-mediated pathways in obese individuals (33, 34). TLR4/MyD8 signaling results in the production of TNF-α and IL-6 cytokines which are associated with development and progression of NAFLD to NASH and HCC (35, 36). TLR4 stimulation in KCs induces the production of pro-inflammatory cytokines (TNF-α, IL-1, IL-6 and IL-8, chemokines) and profibrogenic factors (TGF-β), which will promote fibrosis by activating HSCs (26, 28). Activated Lipopolysaccharide (LPS)/TLR4 signaling in HSCs, stimulates production of chemokines, which further recruit KCs. A vicious cycle of unrestricted activation of HSCs by KCs-derived profibrogenic cytokine TGF-β is established, which leads to development of liver fibrosis (37–39). Thus, activation of TLR4 in HSCs has been suggested to be the main mediator of fibrosis and cirrhosis, by initiating collagen production (26, 40). KCs induce fibrogenesis by secreting proinflammatory and profibrogenic cytokines, whereas HSCs mainly produce extracellular matrix in the fibrotic liver (40, 41). LPS/TLR4 and TLR2 signaling has been suggested to be involved in hepatic inflammation-fibrosis-carcinoma (IFC) sequence, which is linked to viral hepatitis. LPS/TLR4 signaling induces anti-viral responses, inflammation, steatosis, fibrosis, and hepatocarcinoma, as well as hepatic fibrosis-mediated portal hypertension, which leads to bacterial overgrowth and intestinal permeability (42).

Zhang et al. investigated the role of TLR4-mediated innate immunity in pathogenesis of CHB in NAFLD subjects and the effect of TLR4 signaling on HBV replication. The TLR4/MyD88 signaling pathway was demonstrated to be activated in the HBV-transgenic mice with NAFLD and HepG2.2.15 cells with SA-induced steatosis and contributed to inhibition of HBV replication (34). It was suggested that increased LPS and free fatty acids (FFAs) in HBV transgenic mice with NAFLD, were sensed by TLR4, stimulating its signaling pathway which results in production of anti-viral cytokine IFN-β and HBV DNA reduction. IL-6 and TNF-α cytokines, which are also induced by TLR4 signaling, have been shown to inhibit HBV replication. Thus, the increased TLR activity in NAFLD, under the effect of fatty acids, seems to have a positive impact on the HBV infection course, by possibly controlling HBV replication (34). Hu et al. developed an HBV- immunocompetent model to investigate the interplay between HBV infection and fatty liver. They showed that hepatic steatosis can be associated with significantly decreased serum levels of HBeAg, hepatic HBcAg and HBsAg, HBV DNA, and pgRNA, indicating a possible positive effect of fatty liver on HBV infection course, related to inhibition of HBV replication and proliferation (43) (**Table 1**).

**Table 1:** Mechanistic role of NAFLD-HBV interplay in chronic liver disease progression.

	Mechanistic role/pathways	Effects on HBV infection	Chronic liver disease progression	References
NAFLD	↑TLR4/MyD88 pathway leads to ↑TNF-α, IL-1, IL-6, IL-8, TGF-β→ ↑HSCs activation	inhibition of HBV replication	progression to liver fibrosis, NASH and HCC	(24, 26, 28, 30, 33–42)
	↑TLR5→NF-κβ, MAPK			
	↑TLRs in NASH stage leads to ↑CD8+ T cells and NKT cells	↑anti-viral immune responses, HBV clearance	↓chronic liver injury	(15–21)
	↑Th17, IL-21			
	↑LPS/TLR4 and TLR2 signaling	↑immune responses, inflammation	inflammation-fibrosis-carcinoma (IFC) sequence in viral hepatitis, steatosis, fibrosis-mediated portal hypertension	(42)
	metabolic stress, ↓PGC-1α	↑HBV suppressed immunity	↓HBV-related liver disease progression	(44–48)
		inhibition of HBV replication		
	palmitic acid→ impaired DCs function	↓HBV specific immunocytes	↑severe HBV-related disease progression	(49–52)
		abnormal/insufficient immune responses		
	Metabolic components ALT, FBS, TGL BMI and waist circumference		↑positive correlation with fibrosis/cirrhosis and hepatic steatosis in CHB patients	(53)
CHRONIC HBV INFECTION	Mechanistic role/pathways	Effects on NAFLD	Chronic liver disease progression	References
	HBV DNA transcription, TFs (FXR, RXR,C/EBP, CREB), interaction between TFs of activated immune cells	hepatic metabolism of glucose, lipids, bile acid, and xenobiotics	promotion of hepatic regeneration, inflammation, fibrosis, and neoplastic transformation	(54–62)
	IL-13 leads to ↑TGF-β1, activation/proliferation of myofibroblasts, ↑JAK/STAT pathway→CCL11 production→eosinophil recruitment		Hepatic steatosis and Fibrosis	(63–80)
	G-CSF expression	↓hepatic lipogenic genes, ↑b-oxidative genes, ↓SREBP-1c		
	IL-4 activates macrophage, M2 → breakdown of ECM, ↑MMP-12			
	↑IL-6 by KCs	↑HSCs proliferation and survival		
	[HBx-HNF3b-C/EBPa-PPARa] activates FAB1	↑fatty acid uptake	Hepatic Steatosis	(13, 81–93)
	HBx activates PPARs, PI3K/AKT pathway and LXR/SREBP pathway→activation of FAS, ACC, SREBP-1c, CYP7A1	inhibition of apolipoprotein B secretion, ↑hepatic lipogenesis, oxidative corvension of cholesterol to bile acids, hepatic lipid homeostasis		
	Hbx interacts with TNFR→activation of NF-κβ pathway			
	HBV pre-S1 binds to NCTP-impede bile acid uptake,↑ expression of cholesterol synthesis genes [3-hydroxy-3-methylglutaryl-coenzyme A (HMG-CoA) reductase and LDL receptor]	altered hepatic cholesterol metabolism		

**Figure 1:**
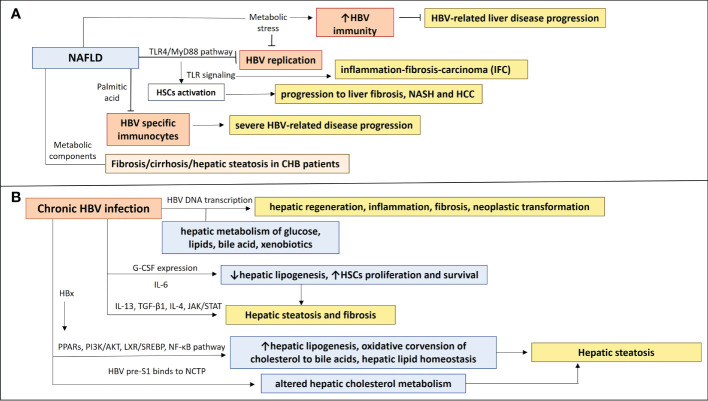
The NAFLD-HBV interplay in chronic liver disease progression. **(A)** NAFLD effects on chronic HBV infection and chronic liver disease progression. Activation of TLR4/Myd88 pathway in NAFLD inhibits HBV replication and induction of TLRs contributes to HSCs activation, leading to inflammation-fibrosis-carcinoma (IFC) and progression to liver fibrosis, NASH and HCC. Production of saturated fatty acid palmitic acid suppresses HBV specific immunocytes, resulting in insufficient immune responses, which could be associated with a more severe HBV-related disease progression. Metabolic components have been implicated in liver disease progression and NAFLD development in CHB patients, as they were correlated with fibrosis/cirrhosis and hepatic steatosis. NAFLD-associated metabolic stress restores HBV-suppressed immunity, preventing from severe HBV-related liver disease progression. **(B)** Chronic HBV infection effects on NAFLD and chronic liver disease progression. Transcription of HBV DNA is related to hepatic metabolism of glucose, lipids, bile acid, and xenobiotics and may play a role in the inhibition or promotion of hepatic regeneration, inflammation, fibrosis, and neoplastic transformation. A differential expression of IL-13, G-CSF, CCL11, IL-6 and IL-4 may be implicated in development of hepatic steatosis and fibrosis in HBV patients, through affecting hepatic lipogenesis and HSCs proliferation and survival. HBx protein can induce PPARs and signaling pathways (PI3K/AKT, LXR/SREBP, NF-κβ), having an impact on hepatic lipogenesis, oxidative conversion of cholesterol to bile acids, hepatic lipid homeostasis and therefore hepatic steatosis. HBV pre-S1 binds to NCTP, leading probably to altered hepatic cholesterol metabolism and hepatic steatosis.

Although HBV has been shown to downregulate TLRs, chronic infection and loss of HBeAg may lead to upregulation of TLR signaling pathways which trigger hepatic inflammation and disease progression (40). The NAFLD-associated metabolic stress, could have a positive impact on CHB, as it can activate the HBV-suppressed innate and adaptive immunity [restoration of antiviral substances, such as endogenous interferons and tumor necrosis factor-α (TNF-α)] which will eliminate HBV virus and prevent severe disease progression. Metabolic alterations in NAFLD could have an effect on HBV replication. In particular, peroxisome proliferator-activated receptor-gamma coactivator 1 alpha (PGC-1α), a key transcription factor in gluconeogenesis, is increased in fasting status and stimulates *in vivo* the HBV DNA replication (44). PGC-1α has been decreased in NAFLD and it has been negatively correlated with NAFLD severity (45). Thus, PGC-1α in NAFLD may lead to inhibition of HBV replication. Accelerated apoptosis of HBV-infected cells has also been attributed to NAFLD effects. Inhibition of autophagy and increased Fas-mediated apoptosis have been remarked in liver samples from NASH patients, indicating that NAFLD could prevent disease progression in CHB patients by eliminating HBV replication and increasing apoptosis of HBV-infected cells, resulting in HBsAg and HBV-DNA clearance (46–48). Another possible effect of NAFLD disease on HBV infection course could be associated with immune abnormalities, which have been observed in NAFLD animal models. Miyake et al. (49) used two well-characterized antigens of HBV virus (HBsAg and HBcAg) to induce adaptive immunity in NAFLD mice. They showed that the saturated fatty acid, palmitic acid, can induce impaired function of DCs, causing down-regulation of HBsAg processing and presentation of DCs. It was also suggested that impaired DC function in NAFLD mice may be attributed to the non-antigen-specific maturation of DCs in these mice, which could be linked with their inability to activate antigen-specific immunocytes (50, 51). This observation along with the fact that NAFLD mice had impaired glucose tolerance could contribute to abnormal or insufficient immune responses, increasing the possibility of a more severe disease course by impeding the HBV clearance in case of a NAFLD-HBV infection co-occurrence (52) (**Figure 1A**) (**Table 1**). It could be suggested that some NAFLD-associated metabolic and immune components may have a positive impact on HBV replication and HBV clearance and thus contribute to prevention of severe HBV-related liver disease progression. However the presence of aggravating factors such as metabolic imbalance and immune dysregulation in NAFLD disease renders the NAFLD-HBV interplay quite complicated, as these factors could enhance the progression to severe liver disease.

## Possible effects of HBV infection on NAFLD disease progression

The pathophysiological mechanisms beyond the association between HBV infection and NAFLD development and disease progression remains unclear. HBV-related metabolic changes, which could lead to NAFLD development have been observed in animal model studies. NAFLD pathogenesis and hepatic steatosis relies on excessive fatty acid uptake and synthesis, which cannot be balanced by oxidation (94). HBV infection could probably promote NAFLD progression to severe hepatic steatosis by affecting lipid biosynthesis. A significant upregulation of lipid biosynthesis gene expression in the liver of HBV transgenic mice, including sterol regulatory element-binding protein (SREBP) 2, ATP citrate lyase (ACLY), retinol-binding protein 1 (RBP1) and fatty acid synthase has been revealed by cDNA microarray analysis (95). Significant changes in long-chain fatty acid and polyunsaturated fat subpathways following HBV infection, along with a significant increase in glycolytic intermediates and glycogen metabolism have also been found. These alterations implied an increased pool of free fatty acid and upregulated glycolysis respectively (96). Rat primary hepatocytes transfected with the HBV genome or HBx have shown major alterations in long-chain fatty acid and polyunsaturated fat subpathways and increased glycolytic intermediates and glycogen metabolism. Thus, HBV infection could have an effect on NAFLD development by promoting significant metabolic changes, associated with NAFLD (97). However, Hu et al. have shown no specific effect of HBV infection in lipid metabolism and insulin resistance in an HBV-immunocompetent and NAFLD-induced mouse model. In particular, there were no increase in plasma and hepatic lipids or cholesterol and changes in plasma glucose and insulin levels in HBV-NAFLD co-occurrence compared with NAFLD group (43). Positive Hepatitis B core antibody (HBc) has been associated with high incidence of cirrhosis, cirrhosis complications and HCC in NAFLD patients (98).

Long noncoding RNAs (lncRNA) could constitute another factor in the interplay between HBV and NAFLD in liver disease progression. LncRNA may have a role in liver inflammation, considering its implication in the regulation of gene expression and various physiological and pathological processes (99, 100). Increased expression levels of lncRNAs have been observed in CHB patients (101). Higher expression levels of lncRNA EXOC7 have been found in liver tissues and serum of NASH patients compared to patients without steatohepatitis, and they were positively correlated with the aggravation of liver steatosis and inflammation (102). Li et al. (103) analyzed expression profiles of lncRNAs and mRNAs in treatment-naive patients with chronic HBV infection and NAFLD. Expression level of long-chain noncoding RNA-metastasis associated in lung adenocarcinoma transcript 1 (MALAT1) was significantly higher in CHB group than NASH group, suggesting that MALAT1 plays an important role in the HBV-infection-related inflammatory response of patients with chronic HBV infection and NAFLD. An mRNA encoding thioredoxin interacting protein (TXNIP), whose expression was significantly upregulated in CHB group and was associated with MALAT1, through competing endogenous RNA, was identified, proposing a potent new regulatory pathway of MALAT1 and TXNIP, called MALAT1- micRNA-20b-5p-TXNIP (103). TXNIP is a protein complex composed of thioredoxin (TRX), reduced coenzyme II (NADPH) and thioredoxin reductase (TRX-R), which has a major impact on regulation of oxidative stress in cells. It may be associated with initiation of inflammatory responses, as it has been found to bind to the nucleotide oligomerization domain-like receptor family and pyrin domain containing 3 (NLRP3) inflammasomes, inducing its activation (104). Activated NLRP3 stimulates NF-κβ signaling pathway, resulting in upregulation of pro-inflammatory cytokines pro-IL-1β, pro-IL-18 (105). Thus, activation of NLRP3 inflammasome by MALAT1- micRNA-20b-5p-TXNIP regulatory pathway may lead to chronic HBV infection-related inflammatory responses, contributing to liver injury process. Liver immune cells, hepatic parenchymal cells, bile duct epithelial cells, and hepatic stellate cells express and activate inflammatory components under the effect of related signals. Activation of NLRP3 inflammatory components is implicated in NASH-mediated inflammatory injury and it may be related with high-mobility group box protein (HMGB), but the mechanism beyond this relationship remains unknown. As an increased ROS production has been found in HepG2 expressing full-length HBx protein, Li et al. (103) suggested that HBx protein-induced ROS in HBV-infected hepatocytes activate NLRP3, by interacting with TRX protein. Activation of NLRP3 leads to high production of IL-1β by KCs of liver tissue. IL-1β mediates the expression of immune-related genes and lymphocyte recruitment to the infection site, initiating inflammation responses which result in liver damage and increased ALT (106). The increased levels of lncRNAs in both CHB and NAFLD diseases could aggravate the tissue liver damage by enhancing inflammatory responses which lead to liver injury.

## Effect of HBV viral load and specific plasma markers on NAFLD progression

An inverse correlation between HBV viral load and liver steatosis and an inverse correlation between HBsAg and fibrosis score have been remarked in some studies (46, 107, 108). We must also consider that transcription of HBV DNA in hepatocytes, is conducted under the effect of various host transcription factors (TFs) and coactivators, including CCAAT/enhancer binding protein (C/EBP), cyclic AMP response element-binding protein (CREB) (54, 55), the hepatic nuclear factor 3 (HNF3)/FoxA and HNF4 (56, 57), farnesoid X receptor (FXR), retinoid X receptor (RXR) (58, 59), peroxisome proliferator-activated receptor (PPAR) α and peroxisome proliferator-activated receptor gamma coactivator-1 (PGC-1) (59, 60). Some of these TFs are implicated in hepatic metabolism of glucose, lipids, bile acid, and xenobiotics and they may play a role in the inhibition or promotion of hepatic regeneration, inflammation, fibrosis, and neoplastic transformation, by interacting with other pro-inflammatory TFs, induced by activated immune cells, such as the nuclear factor kappa-light-chain-enhancer of activated B cells (NF-κB) (61, 62) (**Table 1**).

HBV viral load has not been associated with controlled attenuation parameter (CAP) liver stiffness measurement (LSM) scores in chronic HBV patients. The implication of metabolic components in liver disease progression and NAFLD development has also been shown in CHB patients, as they had a significant positive correlation with fibrosis/cirrhosis and hepatic steatosis (53). Specific plasma markers of CHB, such as CCL11, IL-6, IL-4, IL-13 and G-CSF have been shown to have a significant influence on the CAP and LSM scores independent of metabolic components. A differential expression of IL-13, G-CSF, CCL11, IL-6 and IL-4 among patients at different stages of hepatic steatosis, highlighted a possible role of an inflammatory response in the development of hepatic steatosis and fibrosis in CHB patients. IL-13 has been shown as an independent predictor of the liver steatosis severity (53). IL-13 has been referred to play a role in liver fibrosis, as a component of a T-helper type 2 inflammatory response (63) and activates transforming growth factor 1 (TGF-β1) (64, 65). Stimulation of TGF-β1 gene expression mediates the fibrogenic effects of IL-13, which result in activation and proliferation of myofibroblasts, excessive production of extracellular matrix (ECM) and inhibition of ECM degradation (65–67). IL-13 signaling activates JAK-STAT-6 pathway (68), which results in CCL11 production in smooth muscle cells, an eosinophil chemotactic protein, which recruits eosinophils (69). Hepatic infiltration and activation of eosinophils has been associated with steatosis and fibrosis (70, 71). IL-13 has been suggested to contribute indirectly to HBV-related liver fibrogenesis by upregulating CCL11, which has a significant association with liver fibrosis (72). IL-13Rα2 receptor has been found to be overexpressed in hepatic stellate cells in sinusoidal lesions of the liver of NASH patients (65). Granulocyte colony-stimulating factor (G-CSF) has been inversely correlated with hepatic steatosis (53), as it has been related to the down-regulation of hepatic lipogenic genes and up-regulation of b-oxidative genes (73). G-CSF could ameliorate and improve hepatic steatosis by reducing the expression of SREBP-1c (74), a transcription factor, inductor of hepatic lipogenesis and mobilizing bone marrow cells, which contributes to liver regeneration (75). IL-4 and IL-6 have shown a potent protective effect on liver fibrosis (53). IL-4 has shown an anti-fibrotic effect, by activating alternatively activated macrophage, M2, which breakdown extracellular matrix (ECM), leading to resolution of liver fibrosis, by secreting matrix metalloproteinase-12 (MMP-12) (76, 77). IL-6 acts as pro-inflammatory cytokine and is implicated in liver regeneration and metabolic function (78). IL-6 receptors are expressed on all liver cell types and IL-6 signaling can affect each liver cell type separately. Hepatic KCs produce IL-6, which has been shown to promote proliferation and survival of HSCs (79). However data regarding the role of IL-6 in liver fibrosis are contradictory, depending probably on homeostasis between inhibitory and stimulatory signals during the different stages of liver fibrosis and under the effect of different etiologies of liver fibrosis (80) (**Figure 1B**) (**Table 1**).

## Relationship between hepatic steatosis and HBV infection

The mechanisms beyond the increased fibrosis/cirrhosis in chronic HBV patients under the effect of severe steatosis remain to be elucidated. The interaction between viral factors and metabolic components of inflammation, underlying the NAFLD disease progression has to be investigated. There are contradictory data regarding the relationship between hepatic steatosis and HBV. In HBV infection, Hepatitis B protein X (HBx) is implicated in cellular signal transduction pathways and gene transcription related with cell growth and apoptosis. HBx has been suggested to lead to increased lipid accumulation in the liver, by affecting mitochondrial reactive oxygen species levels and oxidative stress, as it can directly interact with the mitochondrial respiratory chain complex subunit (13). Lipid accumulation is also induced in hepatocytes by HBx/fatty acid–binding protein 1/hepatocyte nuclear factor 3-b (HNF3b), CCAAT/enhancer-binding protein a (C/EBPa), and peroxisome proliferator-activated receptor a axis (PPARa), which activates FAB1 gene transcription. Over-expression of FABP1 increases the rate of fatty acid uptake (13). Elevated serum levels of FABP1 have been remarked in HBV-infected patients and HBx-transgenic mice (13). HBx protein also interacts with the liver X receptor a (LXRα) or tumor necrosis factor (TNF) receptor 1, leading to NF-κβ activation and TNF production, inhibition of apolipoprotein B secretion, and stimulation of PPARg and sterol-regulatory element-binding protein (SREBP)-1c. LXR/SREBP pathway plays a major role in hepatic steatosis, as LXRs contribute to activation of transcription of enzymes related to the synthesis of fatty acids, including the fatty acid synthase (FAS), acetyl coenzyme A acid enzymes (ACC), and SREBP-1c, and upregulation of the expression of CYP7A1, which participates in oxidative conversion of cholesterol to bile acids (81). SREBPs contribute to hepatic lipid homeostasis (82). HBx interacts with LXRa, enhancing its binding to the promoter LXREs of SREBP-1c and FAS, inducing hepatic lipogenesis (83, 84) (**Figure 1B**).

Induction of PPARs is another endpoint of HBx protein activity. PPARs constitute nuclear receptor proteins, playing a major role in energy metabolism and lipid oxidation, as they modulate the expression of downstream genes related to fatty acid-binding (apolipoproteins A1 and A2) and maintain lipid metabolism homeostasis, including fatty acid uptake, binding, and lipid transport (84). HBx can also upregulate PI3K/AKT signaling pathway, which is implicated in regulation of cell growth, proliferation, and differentiation (85) and can activate SREBP (86). HBx could take part in promoting hepatic steatosis *via* activating pro-inflammatory NF-κβ signaling pathway, as HBx interacts with tumor necrosis factor receptor (TNFR) 1 (87). The role of NF-κβ has been highlighted in promotion of hepatic steatosis and insulin resistance (88, 89). Thus, HBx protein modulates the molecular environment for initiation of inflammation and *de novo* lipogenesis (90).

HBV infection has also been associated with the induction of expression of cholesterol synthesis genes, which predispose to liver steatosis [e.g. 3-hydroxy-3-methylglutaryl-coenzyme A (HMG-CoA) reductase and LDL receptor]. An inverse correlation between HBV and NAFLD has also been found. In particular, hepatitis B surface antigen (HBsAg)-positive patients have shown significantly decreased cholesterol levels, whereas increased cholesterol levels were observed in the HBsAg-negative patients (91). HBV infection can lead to an altered hepatic cholesterol metabolism. An increased expression of low-density lipoprotein receptor and 3-hydroxy-3-methylglutharyl-coenzyme A reductase (HMGCR) has been shown in HBV-transfected cells (92). Genes, related to hepatic cholesterol production and uptake, including those encoding SREBP-2, HMGCR and LDL receptor have been highly expressed in HBV-infected humanized mice. This observation could be attributed to pre-S1 domain of the HBV envelope, which by binding to Na+-taurocholate cotransporting polypeptide (NTCP) could impede NTCP-mediated bile acid (BA) uptake and lead to compensatory production and uptake of cholesterol (93). Non-alcoholic hepatic steatosis has been shown to inhibit HBV replication in a HBV-immunocompetent mouse model, as indicated by the reduction of HBV DNA and HBV-related antigens, whereas HBV replication has not been related with altered lipid metabolism in mice (43) (**Table 1**).

## The adipose tissue: A possible linker between HBV, hepatic steatosis and liver injury

Crosstalk between adipose tissue and liver has a major effect on fatty liver disease development. Excessive fat accumulation on adipose tissue, due to obesity or alcohol consumption leads to alterations in adipose tissue endocrine functions. The function of triglyceride storage in adipocytes is disrupted, resulting in lipotoxicity and increased transfer of fatty acids in liver. This could favor the development of hepatic steatosis. Adipose tissue secretes a variety of pro- and anti-inflammatory cytokines termed adipokines, including tumor necrosis factor (TNF)-α, IL-6, resistin, leptin, and adiponectin (109). Adiponectin exerts anti-inflammatory action by inhibiting the synthesis and release of TNF-α from macrophages in adipose tissue (110). The production of adipokines by adipocytes is affected by nutritional status and plays a crucial role in biological functions and some adipokines are also produced by hepatocytes (111). Adipokines could constitute another link between HBV, hepatic steatosis and risk for liver fibrosis and HCC development. It has been speculated that the increased serum levels of TNF-α, resistin, and leptin in obese patients, as well as adiponectinemia may enhance steatosis, inflammation, fibrogenesis, or hepatocarcinogenesis in the liver (112). However the exact mechanisms beyond this association remain to be elucidated.

Adipokines can mediate the progression of liver injury. Leptin has been shown to enhance fibrotic responses to injury (113). The amelioration of adipose inflammation in NAFLD, induced by weight loss or use of thiazolidinediones (TZDs), has been shown to improve liver injury (114). TNF-α and adiponectin have been implicated in NAFLD (115, 116). The adipose tissue dysfunction, characterized by a dysregulated response of adiponectin to fat metabolism and ingestion has been shown to modulate liver injury and cardiometabolic risk in NAFLD (117). Adiponectin is decreased in NAFLD patients compared to healthy controls and physiologically suppresses fatty acid synthesis and promotes mitochondrial β -oxidation. Hepatocyte death and pro-inflammatory responses, that enhance liver injury and progression to fibrosis are also induced by TNF-α activity (114). Reduction of adiponectin levels in liver tissue of NAFLD patients has been suggested to modulate a pro-inflammatory microenvironment, resulting in increased lipotoxicity and promotion of simple steatosis to NASH and fibrosis (118). Adiponectin has been shown to limit pro-inflammatory responses in obesity by limiting IFN-γ and IL-17 producing CD4+ T cells in obesity (119). Roberts et al. have proposed a possible molecular crosstalk between liver and white adipose tissue that could lead to enhanced liver disease progression. In particular, a feed-forward loop between hepatic unconventional prefoldin RPB5 interactor (URI) and cytokine interleukin-17A (IL-17A) has been remarked to promote DNA damage and systemic inflammation leading to NASH and HCC. URI and IL-17A contribute to cross-talk between liver and white adipose tissue, where lipolysis, neutrophil infiltration and insulin resistance occur, resulting in hepatosteatosis and liver injury (120). HBV DNA has been positively correlated with serum adiponectin, which has been shown to decrease in patients with insulin resistance and hepatic steatosis (121). Serum TNF- α and IL-6 cytokines have been increased in HBV patients with significant necroinflammation (122). Wong et al. suggested that the increased production of TNF- α and/or IL-6 could mediate ongoing liver injury. TNF-α enhances survival of HSCs, immune activation and hepatocyte death, promoting liver fibrosis (123), whereas the high production of IL-6 in experimental- induced liver failure has shown to trigger immune suppression and disrupt liver repair, increasing mortality risk (122).

Viral load, HBeAg status and genotypes have not shown any association with insulin resistance and hepatic steatosis. Considering that viral factors are not associated with insulin resistance or pro-inflammatory adipokines, there is probably a separate, independent contribution of adipokines and HBV virus to liver injury (121). Serum leptin levels may be related with fibrosis progression, as they have been positively correlated with hepatic necroinflammation and are higher in cirrhosis stage in CHB patients (121, 124, 125). The potent pro-fibrogenic role of leptin could be attributed to its activity in innate and adaptive immunity, considering that leptin receptors are expressed by DCs, monocytes, neutrophils, macrophages, natural killer (NK) cells, T cells and B cells. Leptin signaling can activate a variety of signaling pathways which regulate cell activation, cell growth, cytokine production and function of immune cells (126). Adipose tissue may also have a potent major effect on HCC development in chronic HBV infection. Non-cirrhotic patients with HBV-related HCC have shown a higher visceral adipose tissue index (VATI), highlighting the VATI as an independent risk factor for HCC (127).

## Possible mechanisms of HBV-NAFLD interplay leading to hepatocellular carcinoma development

Both HBV and NAFLD diseases have been associated with development of liver cancer (128, 129). Considering that hepatocellular carcinoma (HCC) accounts for 93.3% of primary liver cancers (130) and constitutes the fourth, most deadly type of cancer, investigation of early prognostic markers could be of high clinical significance, especially in cases of different liver diseases co-occurrence. A retrospective cohort study by Chan et al. showed that NAFLD is independent risk factor for HBV-associated HCC development and the presence of APOC3 gene polymorphism (related with triglycerides metabolism) increases further the risk for HCC development in CHB patients (131). The mechanisms beyond the interaction between HBV and NAFLD, which contribute to development of HCC are still not elucidated. Each liver disease has its own separate effect on progression to HCC and the possible mechanistic interplay between NAFLD and HBV could probably be illustrated by the co-occurrence of NAFLD and HBV separate activities.

## HBV and NAFLD-mediated signaling pathways related to HCC

The HBV-induced chronic inflammation can lead to mutations in HBV gene and host genome, which can promote the malignant transformation of liver cells, by altering the viral biological behavior and pathogenicity, as well as the homeostasis of cell processes (132, 133). Mutated HBx has been found in HCC cases (134) and the role of HBx in progression of liver carcinogenesis is possibly attributed to its effect on abrogation of cell-cycle arrest and inhibition of apoptosis (135, 136). Hbx has been suggested to lead to increased risk of HCC, by interacting with a variety of proteins and mRNAs, related with signaling pathways and cell processes that regulate protein posttranslational modification, cell-cycle progression and apoptosis. In particular, HBx mutant protein can interact with Bcl-2, a major regulator of apoptosis and farnesoid X receptor (FXR), a major regulator of bile acid synthesis, lipid and glucose metabolism, to promote HCC development (137, 138). HBx can also lead to stabilization of transcriptional oncoproteins Myc and PAX8, by blocking their ubiquitination process (139, 140). The integration of HBV viral DNA into the host genome has been shown to have significant effect on HCC development in patients with occult HBV infection, as it has been associated with changes in tumor suppressor genes, mutations in the p53 ongogene, and genomic instability (141, 142). Thus, HBV can target a variety of ongogenes (143) and regulate the expression of different miRNAs, interfering with multiple signaling pathways, including Wnt, MAPK, STAT, P53, Akt and Notch to promote HCC development (144–146). For instance HBx can promote the proliferation and migration of HCC cells, by regulating expression of miR-1269b in an NF-κB-dependent manner (147). HBx can directly interact with MyH9 protein to activate Wnt/β-catenin/c-Jun signaling pathway, promoting metastasis, proliferation and malignant cell transformation (148, 149). HBx can also aggravate HBV-related carcinogenesis, by activating PI3K/Akt signaling pathway, regulating liver cell proliferation and malignant transformation (150, 151). HBx could enhance tumorigenesis and HCC growth, by inducing the expression of pro-ongogenic MAPK14 and Notch signaling (152, 153). Increased ROS production by HBx, HBs, and HBc HBV proteins (154) constitutes another indirect risk for HCC development. Accumulation of mutated HBs proteins in hepatocytes has been shown to induce endoplasmic reticulum (ER) stress and favor cell growth, by initiating multiple signaling pathways (155, 156). Mutated HBc protein increases production of ROS by stimulating ER stress and activates the NF-κB signaling pathway by promoting the malignant transformation of infected hepatocytes. HBc activity can mediate proliferation, glycolysis, amino acid metabolism and suppression of apoptosis and regulate the Src/PI3K/Akt pathway and blocks the TRAIL/Fas pathway or expression of p53 oncogene (157–160).

The presence of NAFLD in chronic HBV infection could be an aggravating factor in HCC development, as increased hepatic lipid storage leads to lipotoxicity, endoplasmic reticulum stress and reactive oxygen species-mediated DNA damage, which could enhance oncogenesis (161). Abnormal metabolism, dysbiosis of gut microbiota and dysregulation of immune responses have been implicated in NAFLD-mediated HCC development (162). It has been speculated that abnormal alterations in intrahepatic lipid metabolism which may establish insulin resistance and changes in signaling pathways and oncogenes, could lead to inflammation, fibrogenesis and hepatocarcinogenesis (163). Chronic lipotoxicity leads to oxidative and ER stress, which could have a causative role in NAFLD-HCC. Oxidized LDL uptake by macrophages has been shown to stimulate carcinogenetic signaling, by inducing expression of proteins, related to promotion of lipophagy and enhanced lysophosphatidic acid-enhanced Yes-associated protein (YAP) oncogenic activity (163). Similar to HBV virus, NAFLD disease components can interfere with signaling pathways, including signal transducer and activator of transcription (STAT) signaling pathways, which have been associated with HCC development (164, 165). Oxidative hepatic environment in obesity models of NAFLD has been associated with increased STAT-1 and STAT-3 signaling and inactivated STAT-1 and STAT-3 phosphatase T cell protein tyrosine phosphate (TCPTP), promoting hepatic T cell recruitment, NASH, fibrosis and HCC. STAT-1 signaling has been associated with NASH and fibrosis, whereas STAT-3 signaling has been correlated with HCC development (166). The cell cycle-related kinase (CCRK), an androgen receptor-driven oncogene can contribute to hepatocarcinogenesis *via* a signaling pathway dependent on β-catenin and T cell factor (TCF). CCRK has been associated with NAFLD-mediated HCC, by inducing STAT-3 and the mTORC1/4E-BP1/S6K/SREBP1 pathway (167).

The observed microbiome dysbiosis in NAFLD has been also correlated with NAFLD-mediated HCC. Liver inflammation and fibrosis in NAFLD could be attributed to altered bile acid signaling and a persistent immune activation, mediated by increased gut permeability and translocation of lipopolysaccharides (161). NAFLD-HCC patients have shown increased Bacteroides and Ruminococcaceae populations in their gut microbiome compared to patients with NALFD cirrhosis and no HCC. This microbiota profile has been associated with higher levels of cytokines and chemokines (IL- 8, IL-13, CCL3, CCL4 and CCL5) and activated monocytes in blood, indicating that microbiome changes could possibly aggravate the development of HCC, by exacerbating inflammation (168) (**Table 2**).

**Table 2:** Effects of chronic HBV infection and NAFLD on HCC development.

	Mechanistic role/pathways	Effect on HCC development	References
CHRONIC HBV INFECTION	HBsAg→impaired activity of NK cells	enhanced progression to HCC	(169–173)
	inflammatory stimuli and viral proteins→M2-like tumor macrophages	promotion of HCC progression	(174–176)
	HbeAg→upregulation of PD-L1→polarization to M2 protumor subtype		
	HBV-mediated macrophage release of matrix metalloproteinase 9 (MMP9) and IL-23 → blockade of IFNa to IFNAR1	tumor progression and angiogenesis	(177–182)
	HbeAg→MDSCs expansion→dampen T cell function via IDO pathway		
	HbsAg→activation of ERK/IL-6/STAT3 signaling axis→differentiation of MDSCs		
	CCRK→virus-host signaling		
	↓CXCR5+CD4+ Tfh→↓ICOS, IL-10, IL-21→↓Plasmablasts	insufficient anti-tumoral immunity, enhance evasion of tumor cells	(183–187)
	CD8+ T cell, Treg exhaustion, ↑CTLA-4, PD-1 and TIM-3, ↓antibody production		
	↑NLR, Foxp3+ Treg cells	tumor immune escape and metastasis	(188–190)
	↑TGF-β activity→ ↓microRNA-34a→ ↑CCL22→Treg cells		
	↑PD-1 in peripheral blood CD4+ and CD8+ T cells		(191–195)
	↑PD-1, FcRL4 and FcRL5 in HBsAg-specific B cells→ defective antibody production		
	HBsAg suppresses CREB→ ↓TLR9 on B cells→ ↓proliferation of B cells and pro-inflammatory cytokine release		
	↑immunosuppressive type of B cells→ ↓cytotoxic activity of T cells		
NAFLD	↑neutrophils→ ↑matrix metalloproteinase-9	angiogenesis	(196)
	↑PD-L1+ monocytes → ↓tumor specific T cell immunity	insufficient anti-tumoral immunity, poor survival	(197)
	Tregs and MDSCs→immunosuppression of CD8+ T cells and NK cells	tumor immune escape	(161, 198, 199)
	NK dysregulation by IL-15, NK→less cytotoxic ILC1-like phenotype→↓kill cancer cells		
	CCRK-AR signaling→ ↑mTORC1/4E-BP1/S6K/SREBP1 →MDSCs→metabolic reprogramming and immunosuppression	enhance progression to HCC, impaired anti-tumor immune surveillance	(167)
	lipid accumulation → MDSCs→ ↑ROS production, loss of intrahepatic CD4+ T cells		(200, 201)
	Platelet glycoprotein Ibα-mediated aggregation and activation of platelets - KCs	hepatic inflammation and progression to HCC	(202)
	↑linoleic acid→ ↑ROS production in CD4+ T cells→cell apoptosis	impaired anti-tumor immune surveillance	(203, 204)
	exhausted hepatic PD1+CD8+ T cell, ↑CXCR6		
	Th17 cells→ induction of VEGF/E2/PGE2, activation of ongogenic IL-6/Stat3 signalling	tumor growth and angiogenesis	(205–207)
	IgA^+^ plasma cells →PD-L1 mediated suppression of CD8+ T cells, ↓IL-10	inhibition of anti-tumor immunity	(208–210)
	B regulatory cells, producing IL-10/CD40/CD154 signaling pathway		

## HBV and NAFLD-related immune responses and HCC

An altered immune microenvironment is present in both HBV and NAFLD. The tolerogenic status of liver turns into persistent active inflammation, which results in cellular injury and fibrosis, affecting progression to HCC (211). Various immune cells and immune-related markers have been reported in tumor microenvironment, as significant predictors of clinical outcome in cancer patients (211) and dysregulation of hepatic immune cells may have a major effect on hepatocarcinogenesis. Liver is composed of innate and adaptive immune cells, including macrophages, dendritic cells, myeloid-derived suppressor cells (MDSCs), natural killer (NK) cells, CD4+ T, CD8+ T and B cells (212). The observed inhibitory effect of HBV on innate and adaptive immunity may enhance tumorigenesis. Under the effect of a suppressed immune system, the chronic HBV-induced inflammation could evolve in a persistent liver injury and promote the malignant transformation of liver cells. The tumor microenvironment in HBV-associated HCC has been characterized by a more severe immunosuppression compared to the non-HBV associated HCC (213). However, the mechanisms related to this status of HBV-HCC remains to be elucidated. HBsAg has been shown to significantly inhibit the activation and function of NK cells, by inhibiting the expression and activation of STAT3 transcription factor (169). Impaired activity of NK cells has been associated with enhanced progression of hepatitis to HCC (170, 171). HBsAg-mediated increase of monocytes induces expression of higher levels of suppressive cell surface molecules and cytokines (e.g. Tim-3, PD-1 and IL-10) in NK cells of CHB patients (172, 173). Regarding the role of macrophages in chronic HBV infection, inflammatory stimuli and viral proteins can lead to transition of macrophages into M2-like tumor macrophages, promoting HCC progression (174, 175). HBeAg has been shown to induce up-regulation of checkpoint molecular programmed death-ligand 1 (PD-L1) on macrophage, resulting in polarization to M2 protumor subtype, which impairs responses of CD8+ T cell to HBV (176). HBV-mediated macrophage release of matrix metalloproteinase 9 (MMP9) and IL-23 induces the blockade of binding of IFN-α to IFNAR1, which could contribute to tumor progression and angiogenesis (177, 178). MDSCs might affect tumor progression, by favoring immunosuppression, as they have been shown to inhibit T cell proliferation and function and induce Treg cells and tumor-associated macrophages. MDSCs expansion, induced by HBeAg, has been reported in CHB patients, and it has been associated with impaired T cell function, including T cell proliferation and IFN-γ production, as it interferes with indoleamine-2,3-dioxygenase (IDO) pathway (179). HBsAg also activates the ERK/IL-6/STAT3 signaling axis to promote differentiation of MDSCs (180). Cell cycle-related kinase (CCRK) as a regulator of androgen-receptor oncogene, has been implicated in virus-host signaling to promote tumor progression and induce polymorphonuclear MDSCs in HCC (181, 182).

T lymphocytes represent the major regulators of immune responses, which may play a crucial role in tumor development. CD4+ T cells constitute key players in anti-viral and anti-tumor immunity, as they produce cytokines and interact with other immune cells to activate CD8+ T cells and B cells. A decreased number and activity of cytotoxic T cells has been observed in advance stages of HCC and it has been linked with recurrence and poor survival in HCC patients (214). A decreased frequency and activity of specific CD4+ T follicular helper cells (CXCR5+CD4+ Tfh) in HBV-related HCC patients, along with decreased expression of their co-stimulatory molecules (ICOS) and cytokines (IL-10/IL-21), could result in impairment of naïve B cell differentiation into plasmablasts (183). An exhaustion of CD8+ T cell responses, characterized by decreased proliferation and function has been shown in HBV infection, which could further enhance disease progression to HCC, by establishing insufficient anti-tumoral immunity. CD8+ T cells have shown higher expression of inhibitory molecules (CTLA-4, PD-1 and TIM-3) in HBV and HBV-HCC and high expression of programmed cell death protein 1 (PD-1) on HBV-specific T and B cells has led to exhaustion of T cells and decreased production of antibodies (184–186). Exhausted CD8+ T cells and Tregs have been reported in HCC patients, which could further restrict antitumor immune responses (187). HBV-associated progression to HCC has been correlated with increased peripheral blood neutrophil/lymphocyte ratio (NLR) and increased number of Foxp3+ Treg cells (188, 189). In HBV infection, the increased TGF-β activity has been shown to suppress the expression of microRNA-34a, resulting in enhanced production of chemokine CCL22. Increased CCL22 recruits regulatory T (Treg) cells, promoting tumor immune escape and metastasis (190). An imbalance in Th17/Treg ratio has been proposed as indicator of liver cirrhosis process and it has been associated with increased risk for HCC in HBV patients (215). The expression of PD-1 was significantly decreased in peripheral blood CD4+ and CD8+ T cells of patients with HBV-related HCC and it has been associated with accelerated disease progression, compared to patients with HBV or cirrhosis (191). B cells play a crucial role in alleviation of immune responses and disease course in HBV infection (192). HBsAg-specific B cells have shown high expression of inhibitory molecules (PD-1, FcRL4 and FcRL5) and defective antibody production in HBV patients (186, 193). HBsAg can inhibit TLR9 expression on B cells *via* suppressing CREB protein, resulting in decreased proliferation of B cells and pro-inflammatory cytokine release (194). A high frequency of IL-10 producing, immunosuppressive type of B cells, has been remarked in HCC patients, which have been negatively correlated with the expression of granzyme A/B and perforin in CD4+ T cells, leading to suppressed cytotoxic activity of T cells (195).

The NAFLD progress to HCC is accompanied by recruitment and trafficking of innate and adaptive immune cells in liver during inflammation and fibrosis. Accumulated neutrophils in inflamed liver of NASH patients could induce angiogenesis, by promoting the secretion of matrix metalloproteinase-9 (196). Specific PD-L1+ monocytes, which suppress tumor-specific T cell immunity, leading to poor survival have been found in HCC patients (197). Tregs and MDSCs could favor tumor immune escape in NAFLD, as they have been shown to exert immunosuppressive effects on CD8+ T cells and NK cells in NASH (161). Dysregulation of NK cells, probably mediated by IL-15 activity, has been involved in NAFLD progression (198). The observed transformation of NK cells into less cytotoxic ILC1-like phenotype in NAFLD, has been linked with their impaired activity in killing cancer cells (199). The impaired activity of NK cells to control HSCs activity in advanced fibrosis in NAFLD, could further lead to deterioration of liver tissue in NAFLD-HCC patients (216). CCRK-AR signaling has been proved to establish a pro-tumorigenic environment in mice with obesity-associated HCC. Activated CCRK led to induction of mTORC1/4E-BP1/S6K/SREBP1 signaling pathways, resulting in recruitment of MDSCs, which enhance progression to HCC, by initiating metabolic reprogramming and modulating an immunosuppressive microenvironment (167). Lipid accumulation in liver has also been shown to promote recruitment of MDSCs and lead to increased ROS production in NASH mice model (200, 201). The interaction between liver KCs and highly activated platelets, along with platelet glycoprotein Ibα-mediated aggregation in NASH, has been shown to promote immune cell recruitment, which could enhance hepatic inflammation and HCC development (202).

In the context of adaptive immunity, the dysregulation of lipid metabolism in NAFLD has been associated with a selective loss of intrahepatic CD4+ T cells which further could lead to progression to HCC, highlighting a possible link between abnormal lipid metabolism and impaired anti-tumor immune surveillance. Progression of NAFLD to HCC has been shown to be delayed by the *in vivo* induction of hepatic CD4+ T cell population, mediated by ROS blockade (217). Brown et al. proposed a mechanistic role of dysregulated lipid metabolism in HCC development in NAFLD, indicating a major effect of accumulated linoleic acid on CD4+ T cells. Increased lipotoxicity and hepatocyte death induce linoleic acid release, which has been associated with increased production of ROS and CD4+ cell apoptosis (203). The presence of an exhausted, hepatic PD1+CD8+ T cell population, characterized by increased expression of C-X-C motif chemokine receptor 6 (CXCR6) and TNF-α in NASH mice, has been related with increased NASH progression to HCC, by possibly impairing immune surveillance (204). Th17 cells constitute another cell population, which have been positively associated with human fatty liver-associated HCC (205). Infiltration of Th17 in tumor microenvironment has been shown to promote tumor growth and angiogenesis, through induction of angiogenic factors (vascular endothelial growth factor/VEGF and prostaglandin E2/PGE2) and activation of oncogenic IL-6/Stat3 signaling (206, 207). An increased and highly active CD20+ B cell population has been observed in NAFLD patients (218). The number of tumor‐infiltrating B cells has been associated with tumor progression in HCC (208). Accumulated IgA^+^ plasma cells in NASH-related fibrosis have been shown to suppress CD8+ T cells *via* programmed cell death ligand 1 (PD-L1) and IL-10 expression, contributing to development of HCC in NAFLD (209). IL-10 producing, B regulatory cells have been shown to promote HCC growth, through direct interaction with tumor cells, mediated by CD40/CD154 signaling pathway (210) (**Table 2**).

## Discussion

As both chronic HBV infection and NAFLD diseases can lead to chronic liver injury, and result in severe hepatic complications, HBV and NAFLD co-occurrence raises high concerns regarding the clinical management of patients. Dysregulated fatty acid metabolism and lipotoxicity in NAFLD disease may initiate activation of signaling pathways that enhance pro-inflammatory responses and disrupt hepatocyte cell homeostasis, which could either promote liver injury and progression of NAFLD disease to NASH, fibrosis and HCC or affect HBV replication and immune encountering of HBV virus during CHB. The metabolic dysregulation has been associated with increased cell stress and lipotoxicity in NAFLD, leading to trigger of inflammation, recruitment of immune cells in liver and hepatocyte death. Specific nuclear receptors, expressed by immune and liver parenchymal cells, are activated by inflammatory and stress stimuli and initiate signaling pathways related to fibrogenesis and hepatic steatosis (219). Fatty liver has also been linked to HBV replication, as patients with HBV-NAFLD co-occurrence have shown decreased viral replication (107). Chronic HBV infection is suggested to have an influence on metabolic changes, which could lead to NAFLD development and the HBV-induced inflammatory responses and molecular pathways may constitute an aggravating factor in hepatic steatosis development. However the role of HBV-NAFLD interplay in hepatic steatosis development might be more complicated as Xin et al. have proposed two opposite effects of HBV infection on steatosis. Specifically, CHB could be correlated with decreased risk of hyperlipidemia and lower prevalence of steatosis, probably due to an elevated serum adiponectin level and increased hepatic lipid accumulation could be induced by HBx overexpression and the observed genetic susceptibility to fatty liver in CHB patients (220). We must also consider the significance of the immune homeostasis imbalance which characterizes both HBV infection and NAFLD and its implication in liver disease progression to HCC. The disruption of immune cell function, which can be either induced by the dysregulated lipid metabolism in NAFLD, or the HBV-mediated immunosuppressed microenvironment, could impair the anti-tumor immunity and result in liver cancer progression. The presence of fatty liver has been associated with increased risk for HCC development in CHB patients (11). Further experimental studies are required to elucidate the exact mechanisms beyond the possible interaction between the inflammatory components and signaling pathways of both HBV and NAFLD and their impact on liver pathophysiology. Some studies have focused interest on clinical impact of targeting specific molecules, which are implicated in molecular signaling and immune responses on liver disease progression and response to treatment. Liu et al. have shown that serum IL-21 levels were increased at 12 week of HBV treatment, predicting early anti-viral response in patients with CHB and NAFLD (221). A phase I clinical study has investigated the therapeutic effect of OPB-111077, a novel STAT3 inhibitor, in patients with advanced hepatocellular carcinoma, which was proved to be well-tolerated (222). Restoration of miRNAs in HCC has shown to suppress tumor progression and improve chemosensitivity (223, 224). Zhong et al. suggested that blockade of T cell co-inhibitory receptor TIGIT combined with HBsAg vaccination in a mouse model of HBV-related HCC is able to recover immune homeostasis by reversing hepatic CD8+ T cell tolerance to HBsAg (225). Thus, investigation of the molecular background beyond the HBV and NAFLD co-occurrence is of high clinical significance in the context of designing effective therapeutic targets which will prevent or ameliorate the hepatic complications.

## Author contributions

ET, SA and CT conceived and coordinated the study. ET and SFA did the literature search and analysis and wrote the manuscript.CT, KT, SA and MM were responsible for the revision of the manuscript for important intellectual content. ET, CT, KT, MM and SA approved the submitted version of the manuscript. All authors contributed to the article and approved the submitted version.

## Funding

The publication of this article has been financed by the Research Committee of the University of Patras.

## Conflict of interest

The authors declare that the research was conducted in the absence of any commercial or financial relationships that could be construed as a potential conflict of interest.

## Publisher’s note

All claims expressed in this article are solely those of the authors and do not necessarily represent those of their affiliated organizations, or those of the publisher, the editors and the reviewers. Any product that may be evaluated in this article, or claim that may be made by its manufacturer, is not guaranteed or endorsed by the publisher.
